# Correction

**DOI:** 10.1111/cas.15475

**Published:** 2022-09-09

In an article^1^ titled “Circ_100984‐miR‐432‐3p axis regulated c‐Jun/YBX‐1/β‐catenin feedback loop promotes bladder cancer progression” by Liang Tong, Huihui Yang, Wei Xiong, Guyu Tang, Xiongbing Zu, Lin Qi, the following changes in the manuscript has been done:

In Figure 1, six BC cell lines (HT‐1376, HTB9, 253 J, BT‐B, Biu‐87 and 5637) were presented in the article. “T24” was mistakenly wrote as “5637”. The correct figure is shown below.

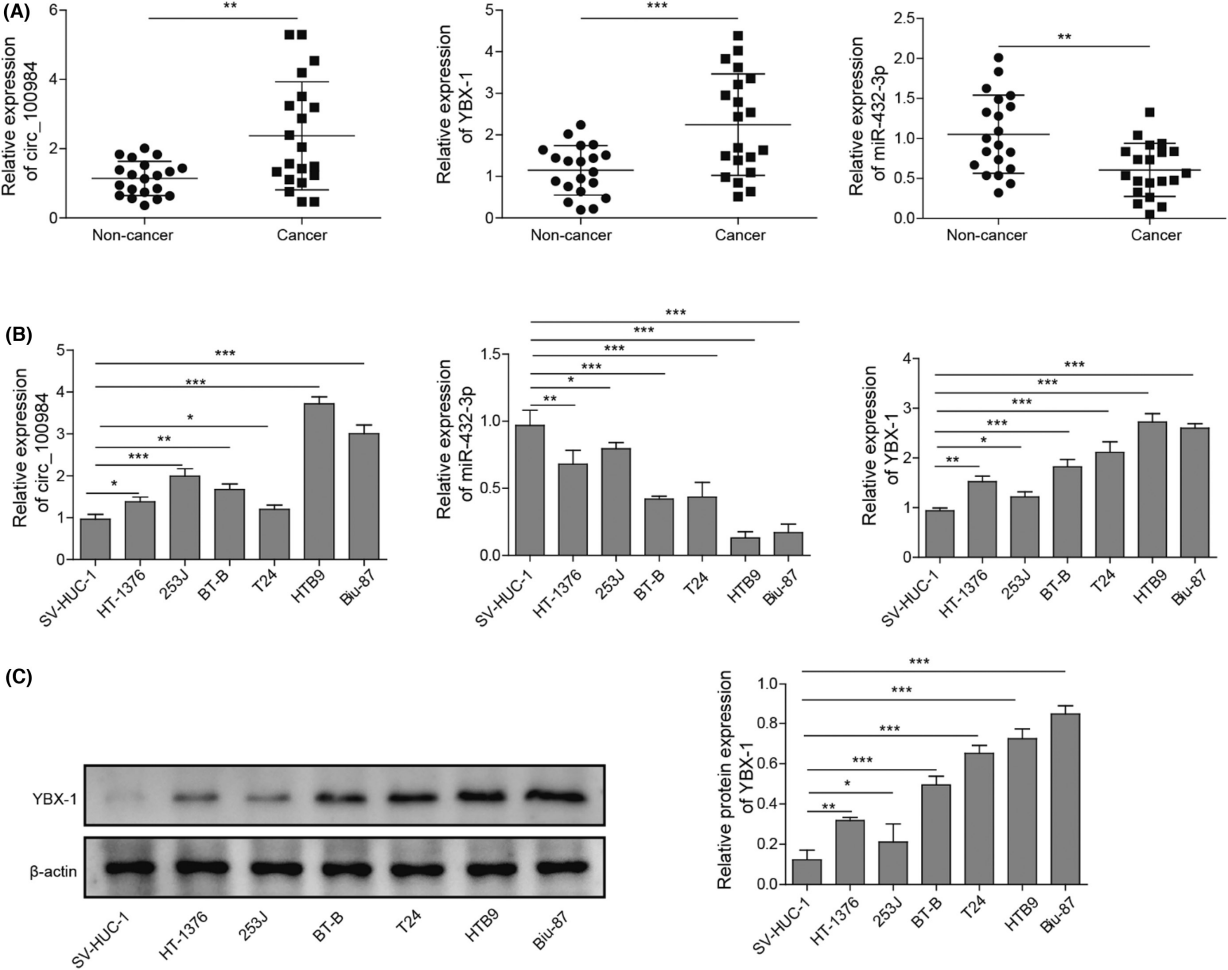

In Figure 2, the cloned image of Biu‐87 cells in Figure 2C was misplaced and is now corrected.

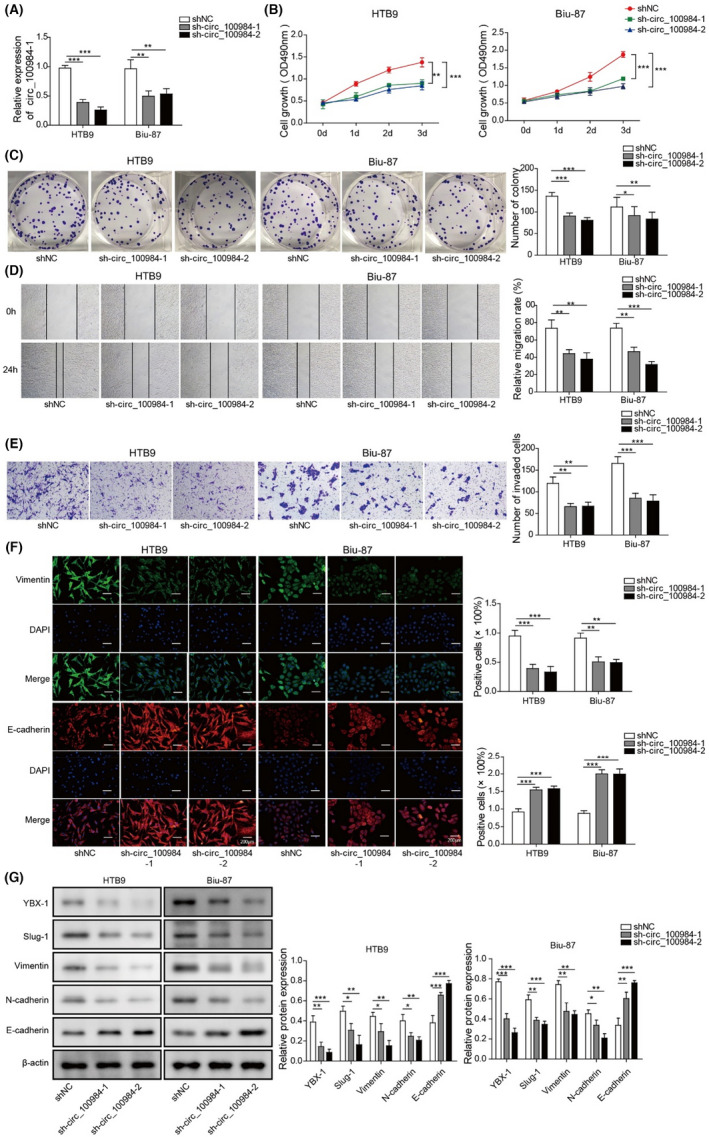

In Figure 3, the cloned image of HTB9 cells in Figure 3C was misplaced and is now corrected.

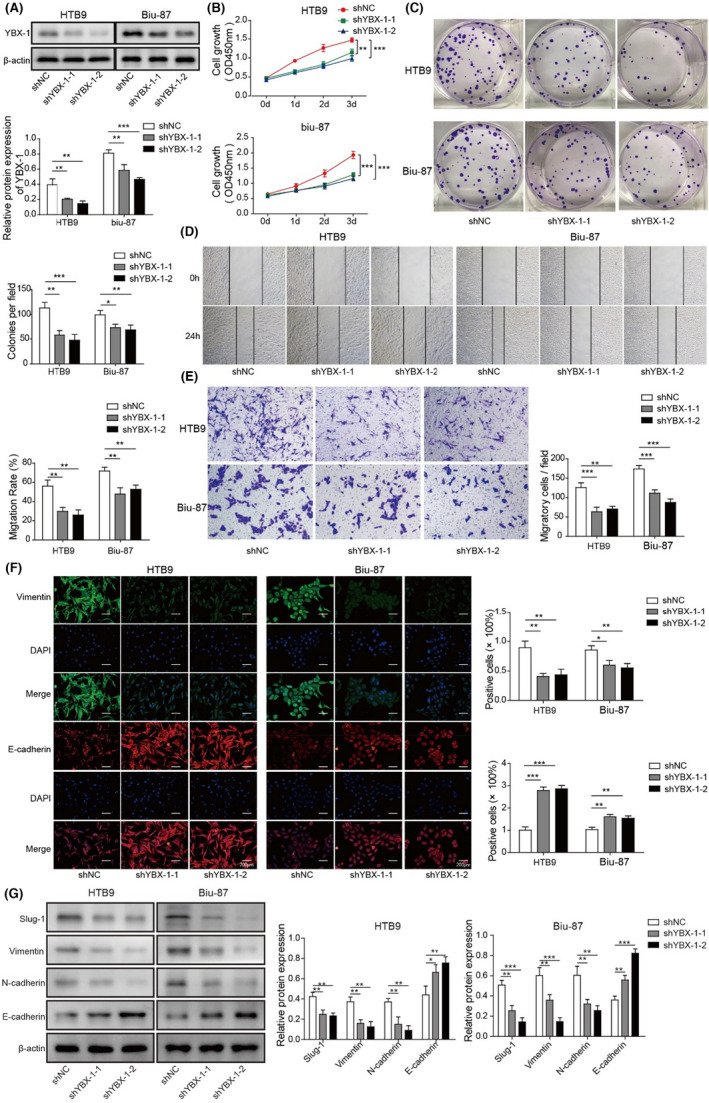

In Figure 7, the image in the Figure 7B was misplaced, the author mistakenly placed the image of Figure 8D in Figure 7B. the corrected Figure 7 is shown below.

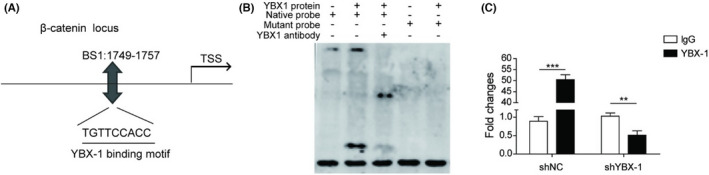

The author apologizes for the error.
